# Low-Temperature Synthesis of Monolithic Titanium Carbide/Carbon Composite Aerogel

**DOI:** 10.3390/nano10122527

**Published:** 2020-12-16

**Authors:** Tingting Niu, Bin Zhou, Zehui Zhang, Xiujie Ji, Jianming Yang, Yuhan Xie, Hongqiang Wang, Ai Du

**Affiliations:** 1 School of Physics Science and Engineering, Tongji University, Shanghai 200092, China; 94niutingting@tongji.edu.cn (T.N.); 1910105@tongji.edu.cn (Z.Z.); 1710867@tongji.edu.cn (X.J.); 1810908@tongji.edu.cn (J.Y.); luwietse@tongji.edu.cn (Y.X.); 1910759@tongjiedu.cn (H.W.); 2 Shanghai Key Laboratory of Special Artificial Microstructure Materials and Technology, Tongji University, Shanghai 200092, China

**Keywords:** titanium carbide, sol-gel, confining template, magnesiothermic catalysis

## Abstract

Resorcinol-formaldehyde/titanium dioxide composite (RF/TiO_2_) gel was prepared simultaneously by acid catalysis and then dried to aerogel with supercritical fluid CO_2_. The carbon/titanium dioxide aerogel was obtained by carbonization and then converted to nanoporous titanium carbide/carbon composite aerogel via 800 °C magnesiothermic catalysis. Meanwhile, the evolution of the samples in different stages was characterized by X-ray diffraction (XRD), an energy-dispersive X-ray (EDX) spectrometer, a scanning electron microscope (SEM), a transmission electron microscope (TEM) and specific surface area analysis (BET). The results showed that the final product was nanoporous TiC/C composite aerogel with a low apparent density of 339.5 mg/cm^3^ and a high specific surface area of 459.5 m^2^/g. Comparing to C aerogel, it could also be considered as one type of highly potential material with efficient photothermal conversion. The idea of converting oxide–carbon composite into titanium carbide via the confining template and low-temperature magnesiothermic catalysis may provide new sight to the synthesis of novel nanoscale carbide materials.

## 1. Introduction

Carbide aerogel is a type of foamed material with three-dimensional nanoporous structure. It combines the advantages of traditional aerogels such as low density, large specific surface area, opening-pore structure and the intrinsic properties of carbides. The crystal structure of typical carbide consists of hybrid bonds including ionic bonds, covalent bonds and metallic bonds, leading to the fundamental characteristics of high hardness, high melting point, wear resistance and conductivity. Therefore, carbide aerogel could be prospectively applied not only in heterogeneous catalysis, heat-resisting materials, superhard additives, fuel cells and sensor elements, etc., but also in high technology fields such as machinery, the chemical industry, carburization and microelectronics, etc. [1,2,3,4,5]. Traditional synthesis methods of carbide include the direct carbonization method [6,7,8], the carbothermic method [9,10,11,12,13,14,15], self-propagation high-temperature synthesis [16,17,18,19,20], the mechanical alloying method [21,22,23,24], the microwave method [25,26], the molten salt method [27,28,29,30] and the plasma method [31,32]. The carbothermic method is widely promoted because it possesses the advantages of low cost, simple equipment, high production efficiency and mass production capacity. However, this method needs high temperature (1200–1500 °C for SiC and 1300–2000 °C for TiC) in an inert or reducing atmosphere, usually leading to the agglomeration and growth of the carbide [9,10,12,13,14,15]. Thus, it is difficult to prepare the ultrafine and nanoscale carbide materials with monolithic appearance.

Only a few works on nanoporous monolithic carbide aerogel have been reported. For example, in 2012 our group developed a facile method for the low-temperature magnesiothermic conversion from C/SiO_2_ aerogel to SiC aerogel via the confining template reaction method at 700 °C [33]. In 2015, a novel low-temperature pseudomorphic transformation of TiC and NbC aerogels was achieved via a solid–gas–solid reaction between the metal and carbon aerogels in aid of iodine catalysts [34]. Kong et al. carried out a series of works about the preparation, control and properties of silicon-carbide-based aerogels, including pure SiC aerogel, mainly by using carbothermal reduction at 1500 °C [35]. These works provide exciting ideas to develop novel nanoporous carbide aerogels.

Previous studies have successfully demonstrated the facile route to carbide-based aerogel. Overall, however, it is difficult to find a delicate balance between the ultrafine structure (high porosity, nanoscale skeleton, high specific surface area, etc.) and rigorous preparation conditions (high temperature and long treating time). By comparison with silicon carbide, titanium carbide normally needs higher treating temperature, indicating the difficulty to maintain both its nanoscale structure and its monolithic appearance. By comparison with the pseudomorphic transformation method, the solid-state reaction is relatively easy to control and scale up. Carbon aerogel is an excellent template because of its low density and high porosity [36,37,38,39,40,41], so it was a good choice for us to increase the strength of the framework structure. Thus, in this paper, we tried to apply the simple low-temperature magnesiothermic conversion method to synthesize titanium carbide in the TiO_2_/C composite aerogel to maintain its nanoporous microstructure and overcome the disadvantage of high temperature and high energy consumption in the preparation process. The main idea was to develop the methods of (1) preparing low-density C/TiO_2_ composite aerogel via a co-gelled process and (2) achieving the magnesium-catalyzed conversion with relatively low temperature and short reaction time. We finally obtained a self-supported monolithic nanoporous TiC/C composite aerogel with low density (339.5 mg/cm^3^) and large specific surface area (459.5 m^2^/g) via a magnesium-catalyzed reaction of only 800 °C and 4 h. This method achieved the goal of controllable structure and adjustable property for the carbide-based aerogels, which may promote the studies of the other novel carbide aerogels with high performances.

## 2. Experimental Setup

### 2.1. Materials

The reagents, including resorcinol (AR, 99.5 wt.%), formaldehyde (AR, 37.0 wt.% in water), acetonitrile (AR, 99.0 wt.%), acetic acid (AR, 99.5 wt.%), nitric acid (AR, 65.0 wt.%), tetra-n-butyl titanate (CP, 98.0 wt.%), ethanol, concentrated hydrochloric acid (AR, 37.0 wt.%) were purchased from Sinopharm Chemical Reagent Co., Ltd. (Shanghai, China). Magnesium powders (1.74 g/mL at 25 °C, 99%) were obtained from Sigma-Aldrich (St. Louis, MO, USA). All reagents were used without further purification.

### 2.2. Preparation of RF/TiO_2_ Composite Gel and Aerogel

**Scheme 1:**
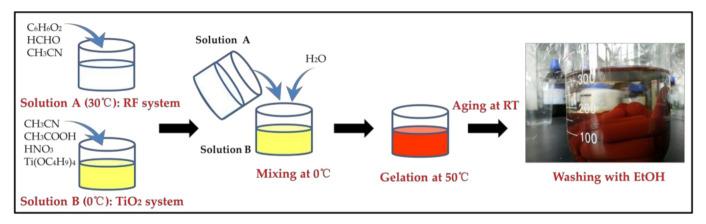
Scheme of the preparation of RF/TiO_2_ hybrid gel.

The synthesis process of preparation is detailed in Scheme 1, displaying the mixture of two parts named as RF system and TiO_2_ system. The key of RF/TiO_2_ composite gel preparation was to successfully compound two monomer gels into one co-gel, so it was very important to control the proportion and concentration of precursors and the reaction condition based on the reaction mechanisms. Firstly, 1.1 g of resorcinol and 2.5 mL of formaldehyde solution (37.0 wt.%) were uniformly mixed with the molar ratio 3:1 in 10 mL of acetonitrile at 30 °C to achieve colorless transparent solution A for the RF system, while 10 mL of acetonitrile, 2 mL of acetic acid, 150 uL of 65.0 wt.% nitric acid and 4 mL of tetra-n-butyl titanate were uniformly mixed at 0 °C in an ice bath to achieve pale yellow semitransparent solution B for another part. Then, solution A was poured slowly into solution B to achieve a red uniform solution. The pH value was in the range of 1~3. Here we determined the molar ratio of the carbon element and the titanium element at the appropriate value 7.6 according to the initial experiments. It must be noted that the mixed solution was stirred at 0 °C because of the temperature sensitivity of tetra-n-butyl titanate. Four milliliters of distilled water could be added to promote the hydrolysis of tetra-n-butyl titanate. After stirring for 30 min, the mixed solution was transferred to a proper sealed container (such as plastic tubes) and gelled at 50 °C within 2 h. The color turned opaque dark red. The wet hybrid gel was placed at room temperature (RT) and aged for 24 h, then removed and repeatedly substituted by ethanol every 6 h for 6 times in order to remove water and residual chemicals. The gel was cut into small slices with a thickness of less than 2 mm, which was beneficial for the magnesiothermic catalysis. At last, they were dried by CO_2_ supercritical fluid to obtain red-brown RF/TiO_2_ aerogels.

### 2.3. Conversion of RF/TiO_2_ Aerogel into TiC/C Composite Aerogel

The RF/TiO_2_ aerogel was placed in a high-temperature carbide furnace under flowing nitrogen (250 mL/min) at 800 °C for 2 h with a heating rate of 3 °C/min to completely carbonize it, so as to obtain black C/TiO_2_ aerogel. Subsequently, C/TiO_2_ aerogel was subject to low-temperature magnesiothermic reduction and acid treatment to be converted into TiC/C composite aerogel. In a typical procedure, 100 mg C/TiO_2_ aerogel and 110 mg magnesium powders were sealed in a steel ampoule with an inner volume of 80 cm^3^ in a glove box full of argon. The magnesium was separately dispersed next to the samples. Then, the ampoule was pushed into a tube furnace under flowing argon (120 mL/min). The temperature was increased to 800 °C and maintained for 4 h. It should be noted that the thickness of the samples was controlled less than 1 mm due to the penetration depth of magnesium vapor. After it cooled to ambient temperature, the ampoule was opened under an inert atmosphere to avoid oxidation. The collected samples were immersed in 10 mL ethyl alcohol. To remove the redundant Mg and the by-product MgO, the corrosion treatment was carried out for 6 h by adding the mixed solution of 10 mL ethyl alcohol, 1.6 mL distilled water and 2 mL concentrated hydrochloric acid (HCl). Then, the purified samples were repeatedly washed by ethyl alcohol every 2 h for more than 4 times and dried by CO_2_ supercritical fluid.

### 2.4. Characterizations

The phase structure of the sample was analyzed by a Model Rigaku D/Max-RB powder X-ray diffractometer (Rigaku, Tokyo, Japan) to analyze its diffraction pattern and obtain the information such as the composition and the structure of the atoms or molecules inside the material. Cu target $K_{\alpha }$radiation (λ = 0.15406 nm) was adopted in the test, with the working voltage of 40 kV and current of 0.04 A. The scanning step was adopted with the step length of 0.08° and the scanning range of the diffraction angle of 10°~80°. Raman spectra were detected at 514 nm excitation using a HORIBA Jobin-Yvon HR800 Raman system (HORIBA Jobin-Yvon, Paris, France) at room temperature. X-ray photoelectron spectroscopy (XPS) was examined by Thermo ESCALAB 250XI (Thermo Fisher Scientific, Waltham, MA, USA). The morphology of the sample was collected by a Philips XL30FEG scanning electron microscope (Royal Philips Electronics, Amsterdam, The Netherlands) with the acceleration voltage of 10 kV; the sample was subject to gilding before observation. Composition analysis was carried out on the samples by an Oxford energy-dispersive X-ray (EDX) spectrometer (Oxford Instruments NanoAnalysis, Concord, MA, USA). Transmission electron microscopy (TEM) was conducted with a Model JEM-2100 electron microscope (JEOL Corp, Tokyo, Japan) made by Japanese JEOL Corp. operating at 200 keV. Nitrogen adsorption–desorption isotherms were measured at liquid nitrogen temperature (77 K) by a Quantachrome Autosorb-1MP analyzer (Quantachrome, Boynton Beach, FL, USA) after the samples were degassed in a vacuum at 150 °C for at least 12 h. The specific surface area was calculated by Brunaur–Emmett–Teller (BET) method.

## 3. Results and Discussion

### 3.1. Appearance and Structural Characterization

**Figure 1:**
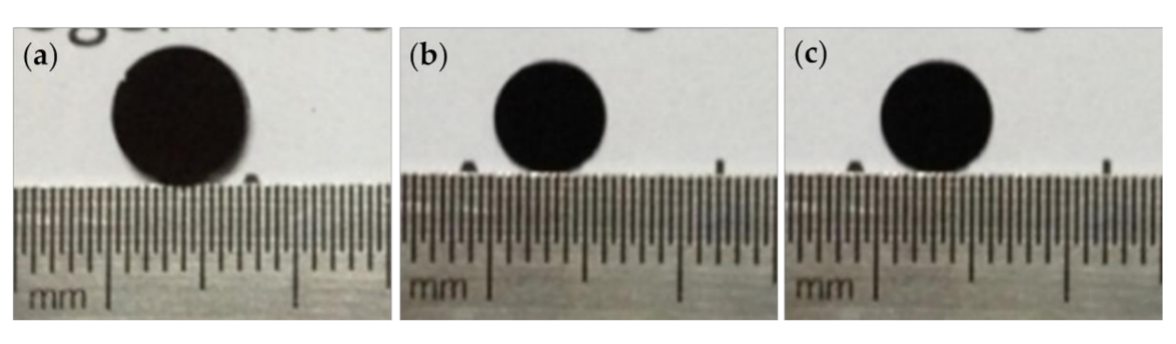
Appearances of samples of (**a**) RF/TiO_2_ aerogel, (**b**) C/TiO_2_ aerogel and (**c**) TiC/C composite aerogel subject to magnesiothermy catalysis, HCl treatment and CO_2_ supercritical treatment.

Figure 1 shows the appearances of the three samples of RF/TiO_2_ aerogel, C/TiO_2_ aerogel and the objective TiC/C composite aerogel. Because all of the samples we obtained had monolithic appearances of regular cylinders, we measured the mass, the diameter and the thickness directly to calculate the density (weighting method). Table 1 lists their average values of densities, diameters and linear shrinkage ratios after repeated measurements. According to Figure 1, the volume of C/TiO_2_ aerogel shrunk slightly after carbonization, while the TiC/C composite aerogel had no obvious shrinkage and maintained a monolithic appearance similar to its original template.

**Figure 2:**
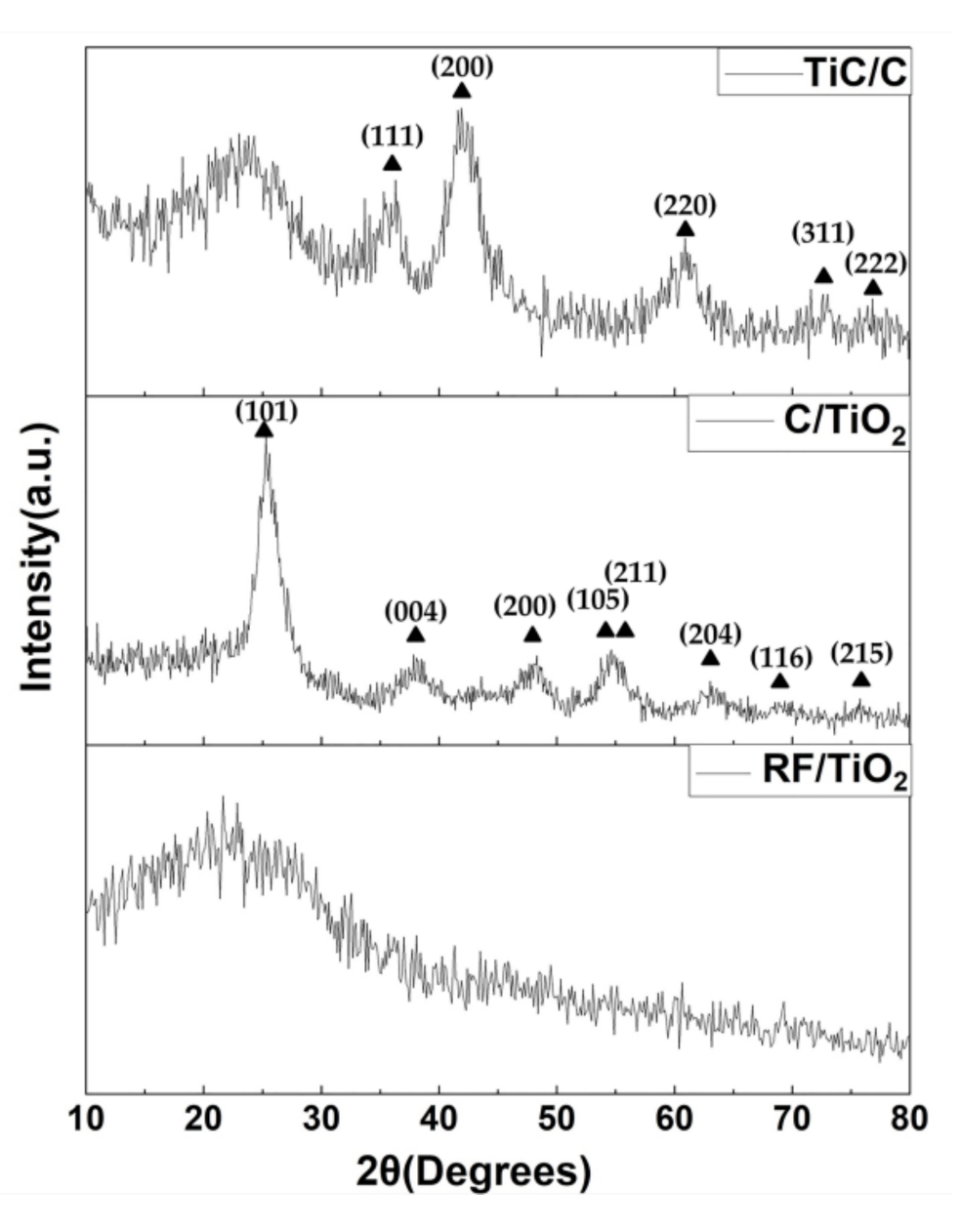
XRD spectra of RF/TiO_2_ aerogel, C/TiO_2_ aerogel and the objective product TiC/C aerogel.

The XRD analysis results of RF/TiO_2_, C/TiO_2_ and TiC aerogels are shown in Figure 2. Only one broad peak in the range of 15–30° was found in the XRD spectrum of RF/TiO_2_ aerogel, which indicated the existence of an amorphous substrate. Several obvious diffraction peaks of C/TiO_2_ aerogel were detected and indexed as anatase-TiO_2_ phase (PDF No. 21-1272). However, the diffraction peaks, located at 2θ  = 35.906°, 41.710°, 60.448°, 72.369° and 76.139° in the sample of objective product TiC/C aerogel, corresponded to the typical (111), (200), (220), (311) and (222) diffraction peaks of cubic-TiC phase, respectively (PDF No. 32-1383). Meanwhile, the phase of TiO_2_ disappeared. This indicated that TiC had been generated from the reaction between TiO_2_ and C through the magnesiothermic catalysis reaction. The final sample contained crystalline titanium carbide. The successful conversion of C/TiO_2_ aerogel into TiC/C aerogel could also be further verified in the following SAED (selected area electron diffraction) patterns. In addition, a broad peak of amorphous carbon was also displayed in TiC/C composite aerogel.

**Figure 3:**
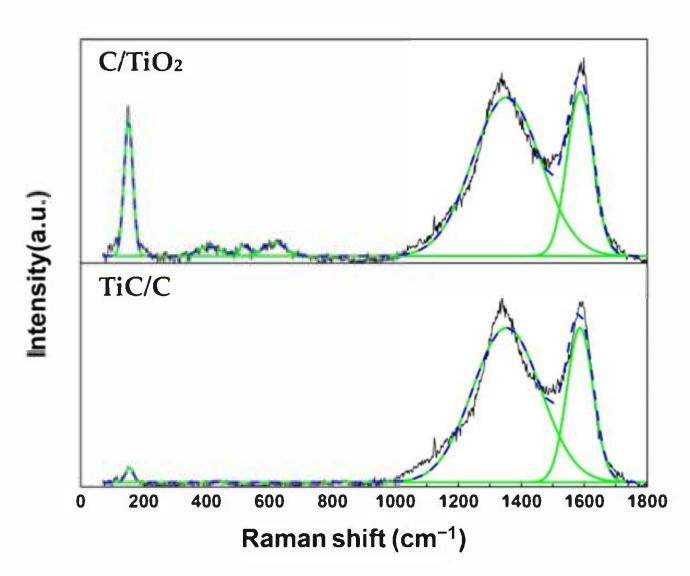
Raman spectra of C/TiO_2_ and TiC/C aerogels.

**Figure 4:**
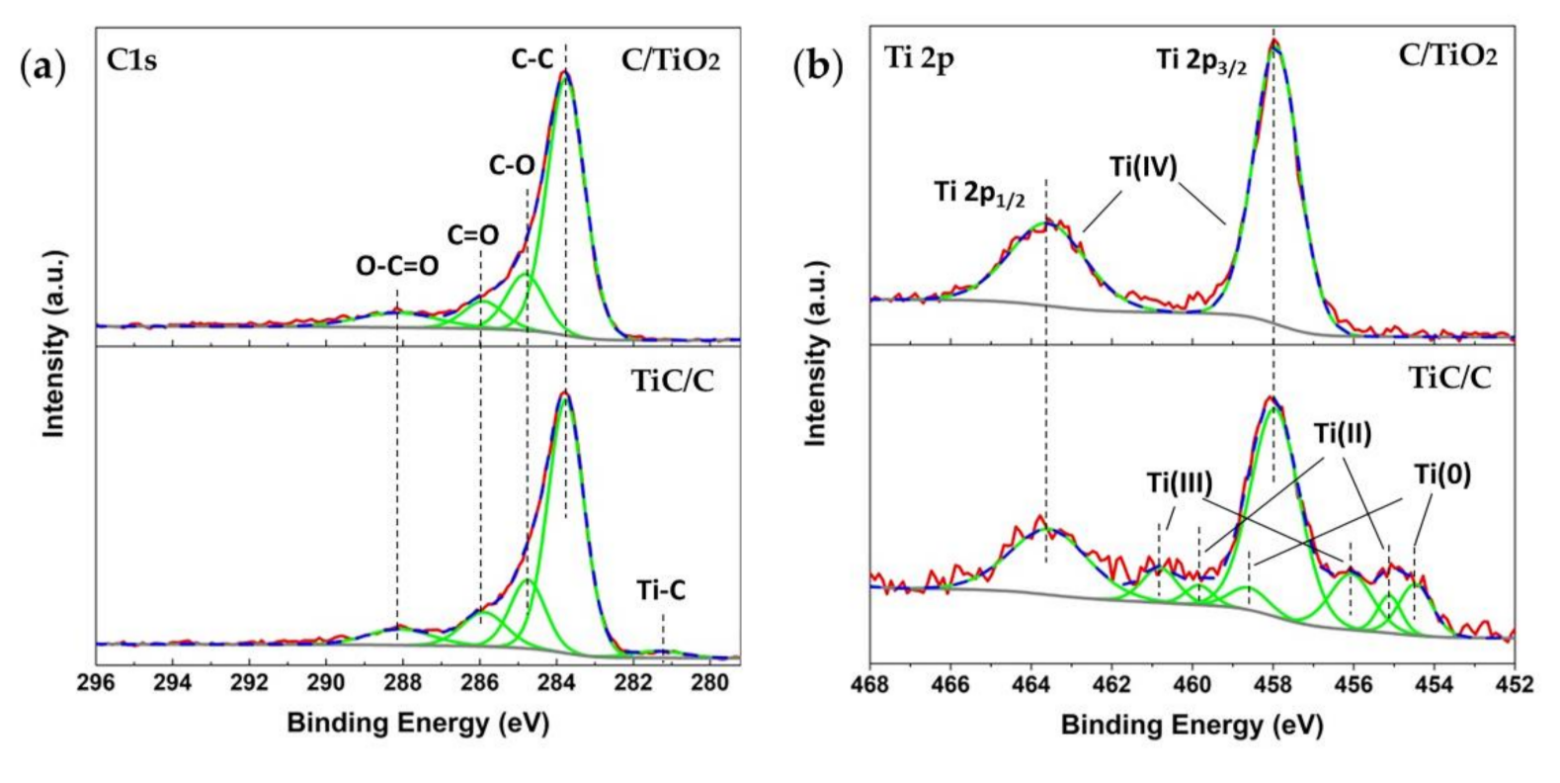
XPS spectra of (**a**) C/TiO_2_ and (**b**) TiC/C aerogels.

Raman spectra of C/TiO_2_ and TiC/C aerogels are shown in Figure 3. For C/TiO_2_ aerogel, the high-frequency branch of the E_g_ mode of anatase-TiO_2_ was detected at 152.1 cm^−1^, as well as B_1g_, A_1g_ + B_1g_ and E_g_ peaks at 411.4 cm^−1^, 518.6 cm^−1^ and 616.7 cm^−1^. However, there was just one weak peak at 155.3 cm^−1^ for TiC/C aerogel assigned to the strongest mode E_g_, which was in accordance with the XRD analysis. The D band and G band of carbon were observed obviously at about 1353.3 cm^−1^ and 1586.6 cm^−1^ for both C/TiO_2_ aerogel and TiC/C aerogel. X-ray photoelectron spectroscopy (XPS) emissions of carbon (1 s) and titanium (2 p) of these two samples are shown in Figure 4 to obtain more detailed information regarding the chemical environment of elements. In both aerogels, it was found that the binding energies (BEs) of C 1 s belonged to C-C, C-O, C=O and O-C=O. Two strong peaks centered at about 457.9 eV and 463.6 eV were ascribed to Ti 2p_3/2_ and 2p_1/2_. As for the TiC/C aerogel, another weak but non-negligible peak could be found at 281.35 eV in the C (1 s) region, which further confirmed the presence of titanium carbide [42,43]. The binding energies at 456.06 eV and 460.81 eV belonging to Ti(III), at 455.12 eV and 459.84 eV belonging to Ti(II), as well as at 454.47 eV and 458.6 eV belonging to Ti(0) were probably caused by the partial reduction of TiO_2_ [44,45].

### 3.2. Morphology and Composition Analysis

**Figure 5:**
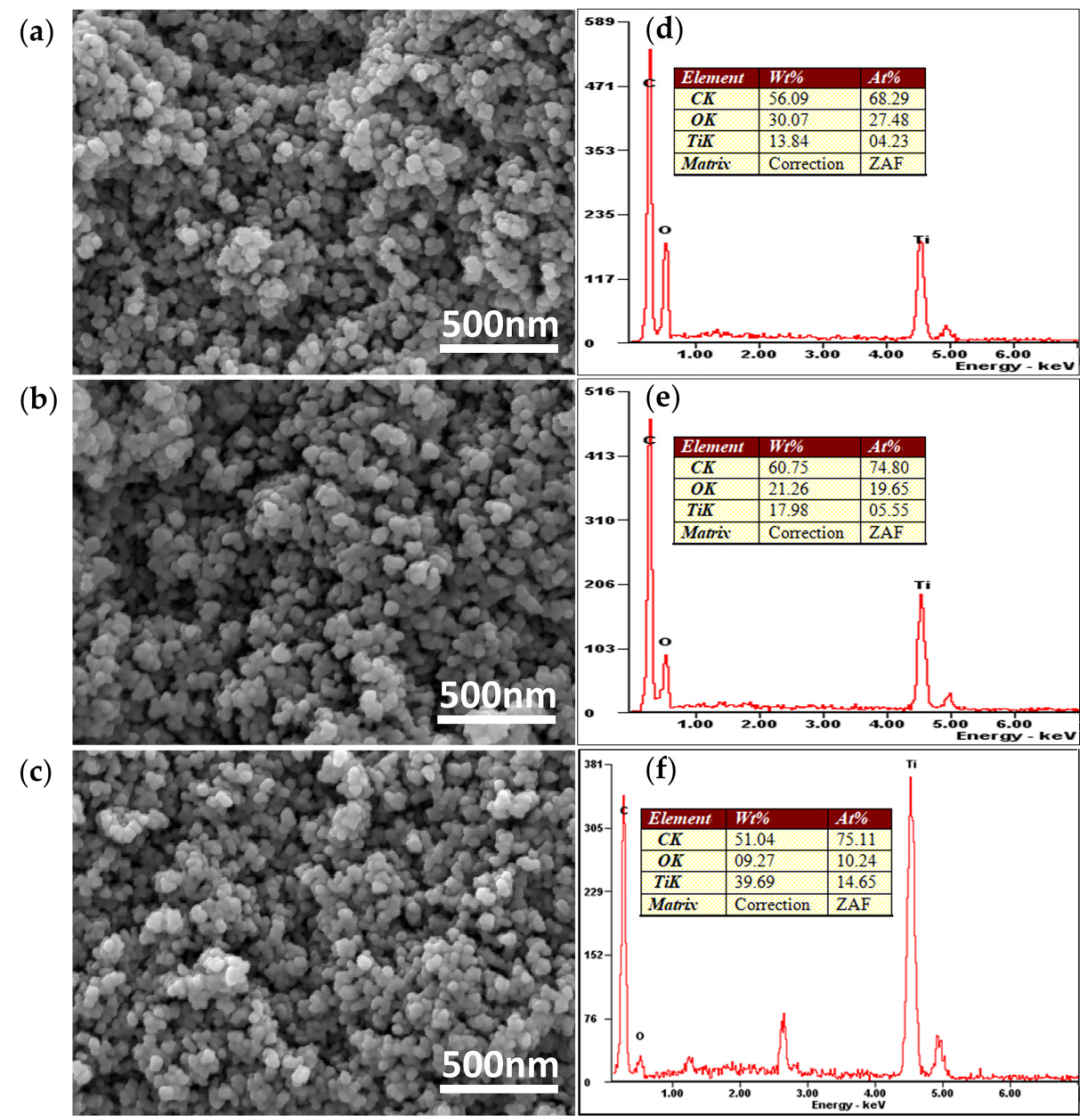
SEM images of (**a**) RF/TiO_2_ aerogel, (**b**) C/TiO_2_ aerogel and (**c**) TiC/C aerogel; the EDX spectra of samples (**d**–**f**) corresponding to (**a**–**c**).

Figure 5 shows the SEM diagrams (a–c) of RF/TiO_2_, C/TiO_2_ and the resulting TiC/C aerogels, respectively. According to the SEM diagrams, these three aerogels all exhibited random nanoporous network structures. The nanoparticles on the framework were distributed uniformly in a globular manner. TiC/C aerogel (Figure 5c) acquired through magnesiothermic catalysis of C/TiO_2_ aerogel (Figure 5b) maintained the porous structure of the original template, which was consistent with the appearances in Figure 1. SEM images indicate that the objective product TiC/C aerogel had no obvious deformation or agglomeration, with a similar spherical particles structure. The results showed that the growth of TiC particles was confined in the microstructure of the original template. Hence, the structure of TiC/C aerogel could be facilely adjusted by controlling the microstructure and shape of the C/TiO_2_ aerogel template.

According to the EDX spectra, relative oxygen content decreased significantly after carbonization and magnesiothermic reduction, which indicated the success of conversion. A small amount of oxygen (Figure 5f) was related to oxidation at the surface of TiC nanoparticles exposed to etching solution or the oxygen absorption at the surface of C nanoparticles. In spite of this, the weight ratio of oxygen and titanium fell from 1.18 to 0.23 after magnesiothermic reduction. Additionally, in order to confirm the content of the Ti element in the resulting TiC/C aerogel, we measured the mass loss after air calcination at 600 °C for 1 h and calculated that the weight percent of Ti was about 30%, which was close to the identification of the EDX spectra.

**Figure 6:**
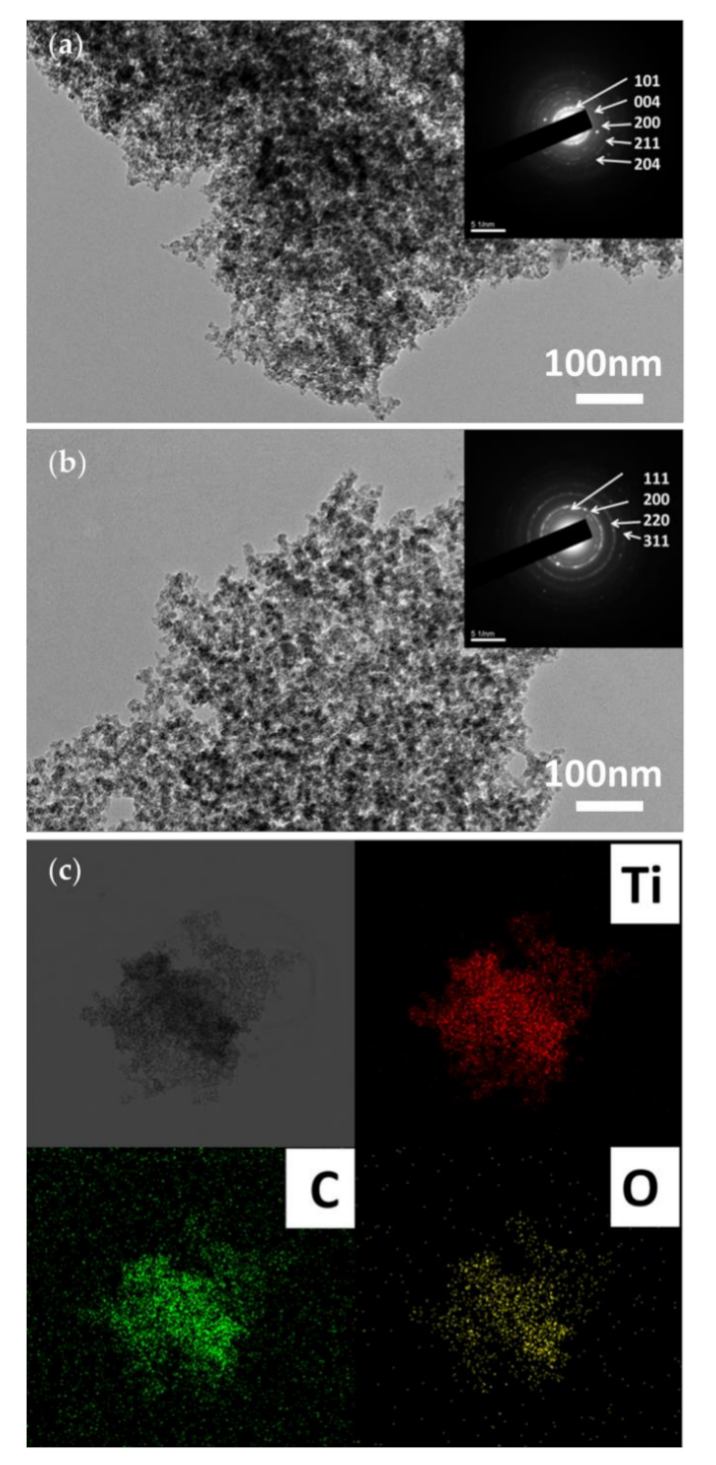
TEM images with SAED inset patterns of (**a**) C/TiO_2_ aerogel and (**b**) TiC/C aerogel; (**c**) mapping results of TiC/C aerogel.

**Figure 7:**
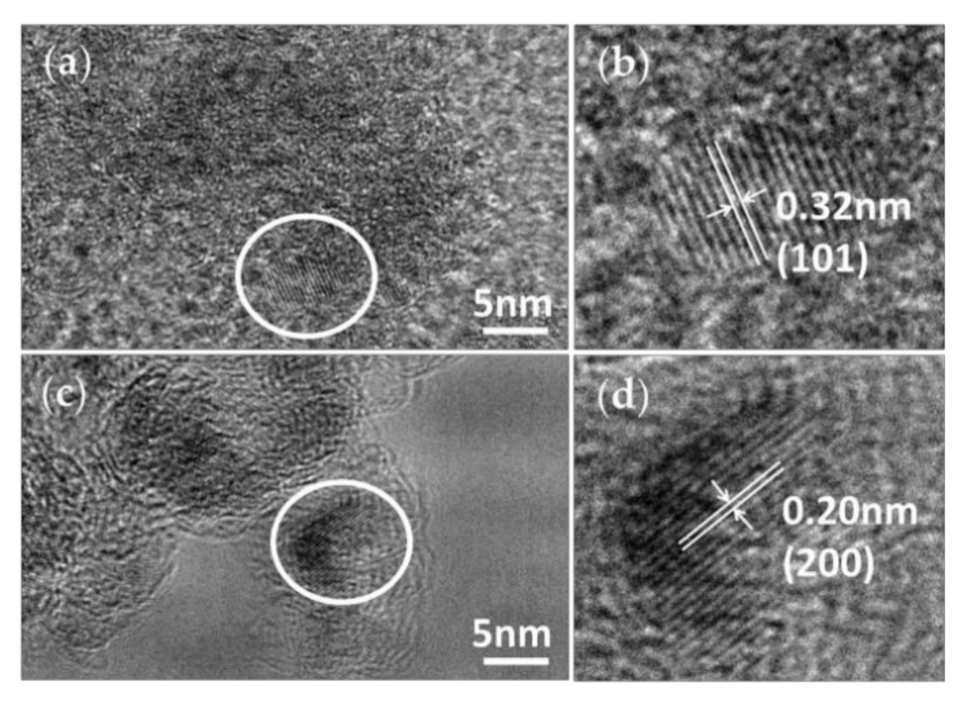
HRTEM images of (**a**) C/TiO_2_ aerogel and (**c**) TiC/C aerogel with (**b**,**d**) the magnified images of the corresponding selected areas in the white circles.

Transmission electron microscopy (TEM) was investigated in order to further understand the microstructure and nanocrystalline structure of TiC/C composite aerogel. Figure 6a,b indicates that the mutually supported framework structures mainly consisted of regular spherical or near-spherical clusters and irregular pores. According to Figure 6c, all the elements had a uniform distribution, while the oxygen content was obviously limited, which was identical to the EDX spectra analysis, indicating a homogeneous TiC distribution. The SAED patterns (insets in Figure 6a,b) show two kinds of different concentric diffraction rings corresponding to the Miller indices of the tetragonal lattice of the anatase-TiO_2_ phase and the cubic lattice of the cubic TiC phase, respectively, which is consistent with the relevant XRD results. The lattice fringes of both aerogels were also easily observed in the high-resolution transmission electron images (HRTEM, Figure 7).

### 3.3. Specific Surface Area Analysis

**Figure 8:**
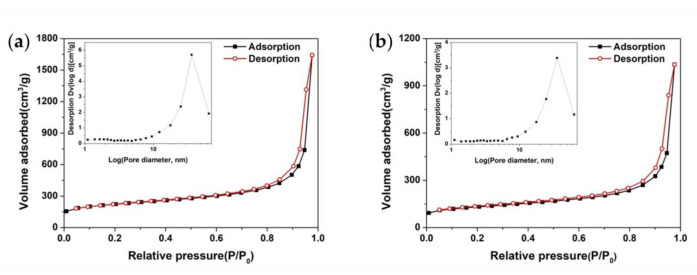
Adsorption and desorption curves and pore size distribution curves of (**a**) C/TiO_2_ aerogel and (**b**) TiC/C aerogel.

The nitrogen adsorption/desorption isotherm (Figure 8a,b) of C/TiO_2_ aerogel and TiC/C aerogel showed Type IV curves, which suggests that they were typical mesoporous materials. They exhibited mixing H1 and H3 hysteresis loops, which are deemed mesoporous systems with cylindrical pores and wedge-shaped pores stacked by nanoparticles. Based on the BJH (Barrett–Joyner–Halenda model) analysis on the desorption curves (shown as insets in Figure 8a,b), pore-size distributions were similar with a single peak around 36 nm. Specific surface area analysis is listed in detail in Table 2. The specific surface area of TiC/C aerogel after magnesiothermic reduction arrived at 459.5 m^2^/g, which was obviously reduced compared to C/TiO_2_ aerogel (780.6 m^2^/g). This may be because the aerogels of the two frameworks reacted to form TiC/C aerogel during magnesiothermic catalysis, which made the original crosslinking framework stack. Another reason may be the dissolution of some impurities containing magnesium during the corrosion process after magnesiothermic catalysis. Due to the reduction in micropore contents, the specific surface area reduced and the average pore size increased.

### 3.4. Photothermal Conversion

**Figure 9:**
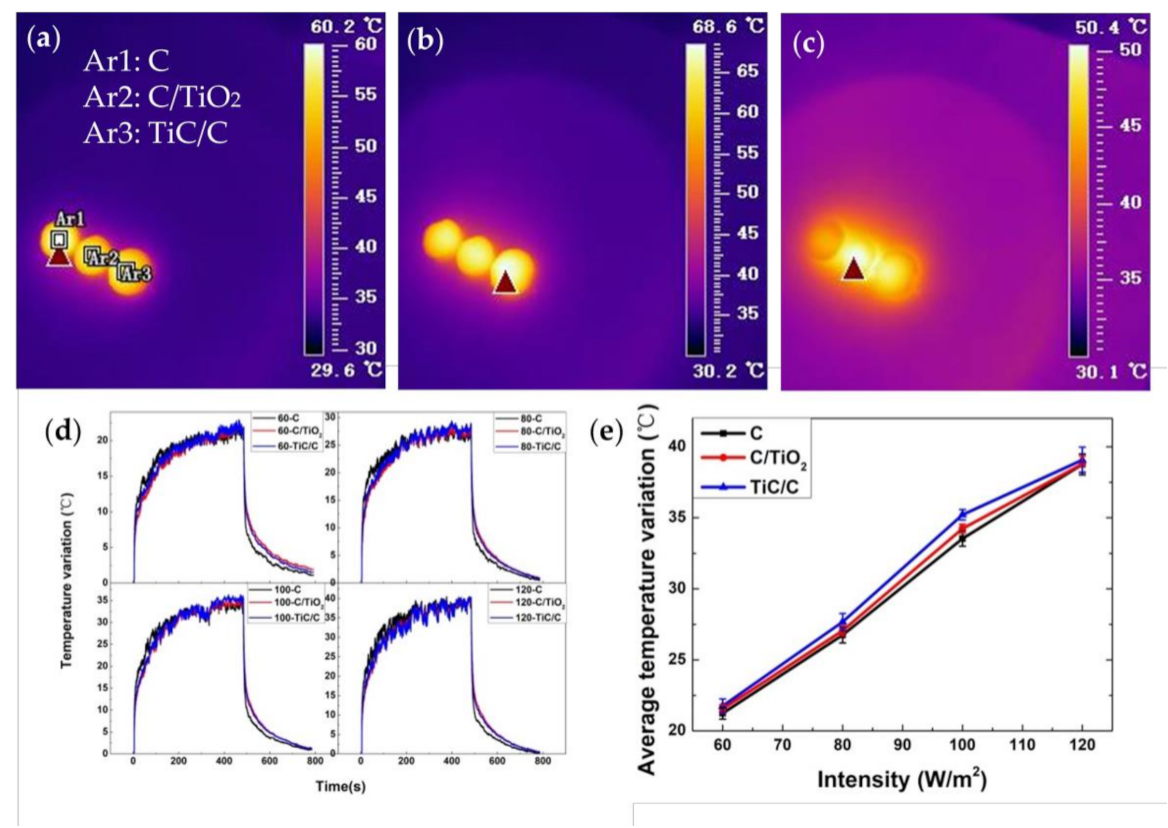
Thermal camera image of three samples after illuminating for 90 s: (**a**, **b**) in the equilibrium state; (**c**) within 6 s after turning off the illumination; (**d**) temperature variation curves of C, C/TiO_2_ and TiC/C aerogels at four different light intensities; (**e**) average temperature variation within 54 s before the ending of illumination at different intensities of three samples.

A simple experiment was conducted to compare the photothermal conversion properties of three different samples (C aerogel, C/TiO_2_ aerogel and TiC/C aerogel). Here, C aerogel was obtained after the corrosion of C/TiO_2_ aerogel with HF. The results of SEM, EDX spectrum and Raman are shown in Figure S1. These three samples were placed under illumination at different light intensities (600, 800, 1000, 1200 W/m^2^) within 8 min. The whole process, including the temperature rise during the illumination and the temperature reduction after the shut-off light source, was recorded using a thermal camera connected to the application of AnalyzIR (Video S1). Figure 9d shows all the temperature variation curves (the average value of a selected area, as shown in Figure 9a) of these three aerogels at four different light intensities. It indicates that C aerogel delivered the fastest response both at the beginning and the ending of illumination. The first derivative curves at the beginning and the ending of illumination within 1 s were compared in Figure S2a,b to further understand the response speeds of different aerogels. We found that C aerogel had the highest sensibility. Nevertheless, the temperature variations of TiC/C aerogel reached the maximum, which exceeded that of C aerogel after a period of illumination (Figure 9b with the highest temperature of the whole area marked). Figure 9e shows the average temperature variation within 54 s before the ending of illumination at different intensities. Meanwhile, the comparisons of temperature variation with C aerogel within 54 s before the ending of illumination are shown amplified in Figure S2c,d. It was obvious that TiC/C aerogel arrived at the highest equilibrium temperature regardless of the light intensity. The opposite results may be due to the difference of thermal conductivity coefficients between C and TiC. According to the cooling part of these curves, TiC/C aerogel dissipated heat slower than C aerogel (obviously observed in Figure 9c), which indicated its coefficient of thermal conductivity was lower. The equilibrium temperature was determined by the combined effect of heat absorption and heat dissipation. Under illumination, these three aerogels were able to convert the energy of illumination into thermal energy. Some thermal energy was absorbed, leading to the rise in temperature, while another part was dissipated. Because of the lower thermal conductivity, TiC/C aerogel had no obvious heat dissipation effect and thus could reach a higher equilibrium temperature. This showed that the TiC/C aerogel was also a highly potential material of efficient photothermal conversion.

## 4. Conclusions

With RF/TiO_2_ aerogel as the template, a novel nanoporous TiC/C aerogel material was prepared by means of a confining template of magnesiothermic catalysis under low temperature (800 °C) and short reaction time (4 h). Firstly, RF/TiO_2_ aerogel was acquired via sol-gel of a compound acid catalysis. After carbonization and subsequent magnesiothermic reaction, the objective product TiC/C aerogel was successfully obtained. It mainly maintained the nanoporous structure of the original material with very integral appearance and ultrafine morphology. Moreover, it had low density (339.5 $\mathrm{mg}/{\mathrm{cm}}^{3}$) and comparatively large specific surface area (459.5${\;m}^{2}/g$). XRD and TEM analyses indicated the formation of cubic TiC phase. The TiC/C aerogel was discovered as a potential material of highly efficient photothermal conversion. This preparation method can be expected to solve the bottleneck problems of preparing nanoscale titanium carbide materials with controllable structure, adjustable composition and density as well as integrated functional structure at low temperature. In addition, this confining reaction method with controllable template structure can expand the study of conversion of carbon/transition metallic oxides to carbides at low temperature, and it is expected to further expand to the study of transition metals such as Hf, Ta and W.

## Figures and Tables

**Table 1:** Densities and diameters of RF/TiO_2_, C/TiO_2_ and TiC/C composite aerogels.

Samples	$\mathrm{Density}\;(\mathrm{mg}/cm^{3})$	Diameter (cm)	Linear Shrinkage Ratio (%)
RF/TiO_2_	234.8 ± 7.6	0.723	--
C/TiO_2_	355.1 ± 10.4	0.582	19.5%
TiC/C	339.5 ± 7.9	0.569	2.2%

**Table 2:** Data related to the pore structures of RF/TiO_2_ aerogel, C/TiO_2_ aerogel and TiC/C aerogel.

Sample	Specific Surface Area (m^2^/g)	Average Pore Diameter (nm)	Pore Size of Maximum Distribution (nm)	Total Pore Volume (cm^3^/g)
C/TiO_2_	780.6	13.05	36.1	2.547
TiC/C	459.5	13.97	35.7	1.605

## Supplementary Materials

The following are available online at https://www.mdpi.com/2079-4991/10/12/2527/s1, Figure S1: (**a**) The corrosion of C/TiO_2_ aerogel with HF; (**b**) Raman spectrum, (**c**) SEM image, (**d**) EDX spectrum of C aerogel, Figure S2. The 1st derivative curves at the beginning (**a**) and the ending (**b**) of illumination within 1 s; (**c**) the comparisons of temperature variation within 54 s before the ending of illumination; (**d**) the difference in temperature variation compared with C aerogel within 54 s before the ending of illumination, Video S1: The whole process of the temperature rise and the temperature reduction.
