# Supplementary material for: Low-Temperature Synthesis of Monolithic Titanium Carbide/Carbon Composite Aerogel
Source: Nanomaterials (Basel). 2020 Dec 16;10(12):2527. doi: 10.3390/nano10122527 (PMC7767110; doi:10.3390/nano10122527)
Supplement: Supplementary file 1 [file nanomaterials-10-02527-s001.pdf]

Supplementary Materials:

Low-Temperature Synthesis of Nanoporous Titanium Carbide/Carbon Composite Aerogel

Tingting Niu [1,2], Bin Zhou[1,2,*], Zehui Zhang [1,2], Xiujie Ji[1,2], Jianming Yang[1,2], Yuhan Xie[1,2], Hongqiang Wang[1,2] and Ai Du [1,2,*]

1 School of Physics Science and Engineering, Tongji University, Shanghai 200029, PR China ; 94niutingting@tongji.edu.cn (T.N.); 1910105@tongji.edu.cn (Z.Z.); 1710867@tongji.edu.cn (X.J.); 1810908@tongji.edu.cn (J.Y.); luwietse@tongji.edu.cn (Y.X.); 1910759@tongjiedu.cn (H.W.)

2 Shanghai Key Laboratory of Special Artificial Microstructure Materials and Technology;

* Correspondence: zhoubin863@tongji.edu.cn (B.Z.); duai@tongji.edu.cn (A.D.)

Preparation of C aerogel

C/TiO_2 aerogel was placed above the corrosive solution mixed with 40 mL EtOH and 1 mL HF for 3 days (as shown in Figure S1a) for the entire corrosion of TiO_2 by HF vapor. It was repeatedly washed by ethyl alcohol every 2 h for more than 6 times and dried by CO_2 supercritical fluid. Figure S1 displays the Raman spectrum, SEM image and EDX spectrum of the final sample after the corrosion treatment. The results indicated that C aerogel was successfully obtained due to the absence of TiO_2, and it maintained the excellent nanoporous network structure.

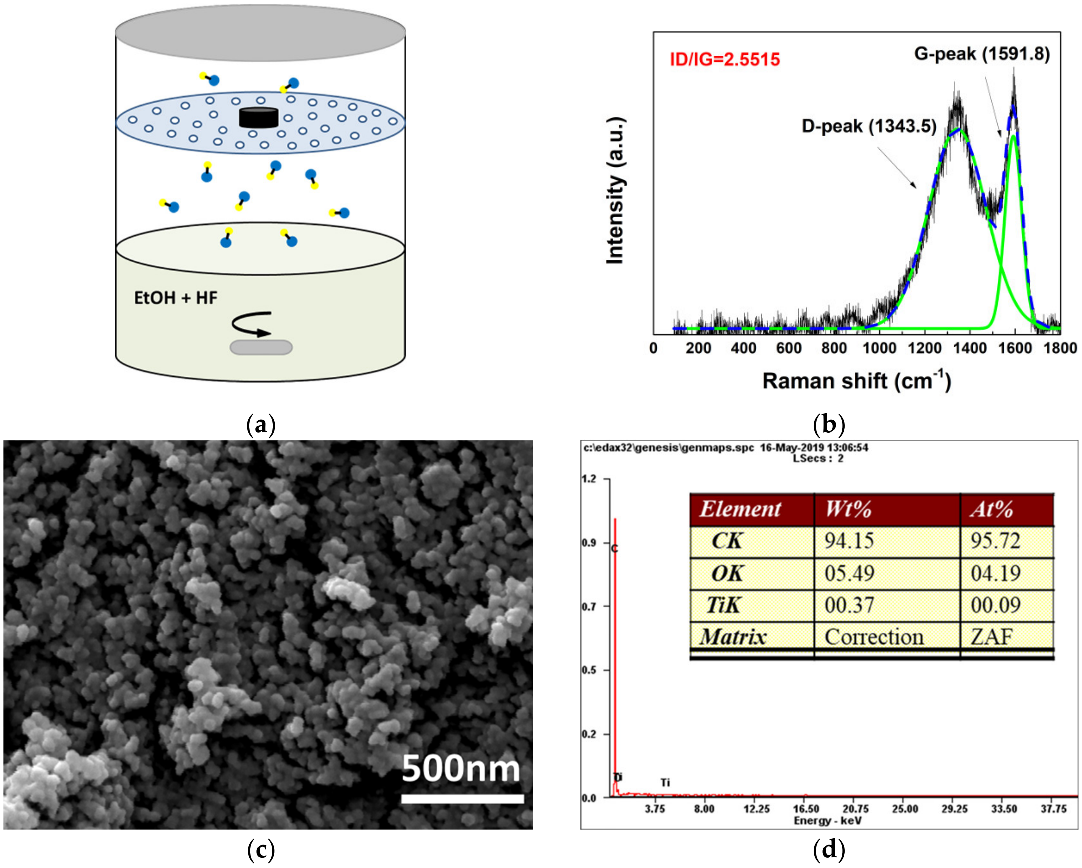

Figure S1. (**a**) The corrosion of C/TiO_2 aerogel with HF; (**b**) Raman spectrum; (**c**) SEM image; (**d**) EDX spectrum of C aerogel.

Photothermal conversion

The following figures show the first derivative curves at the beginning and the ending of illumination within 1 s to compare the response speeds of different aerogels at different light intensities. The temperature variations within 54 s before the ending of illumination were also amplified to examine the equilibrium temperature. In Figure S2d, C aerogel is considered as the reference.

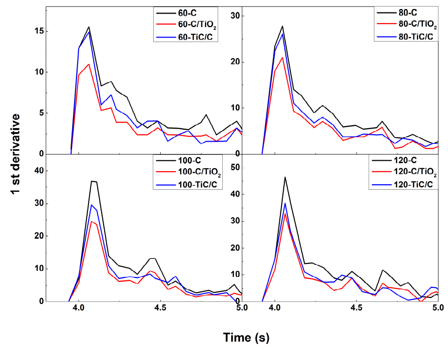

(**a**)

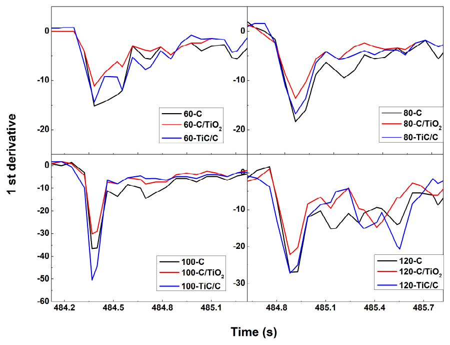

(**b**)

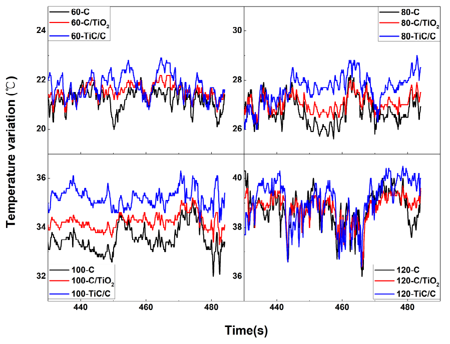

(**c**)

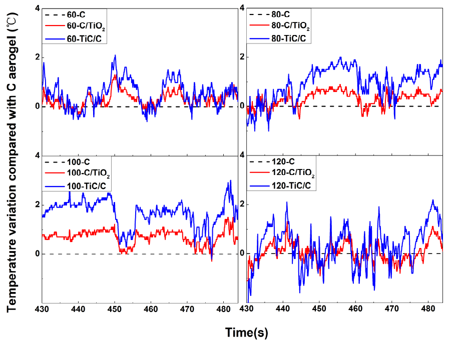

(**d**)

Figure S2. The 1st derivative curves at the beginning (**a**) and the ending (**b**) of illumination within 1 s; (**c**) the comparisons of temperature variation within 54 s before the ending of illumination; (**d**) the difference of temperature variation compared to C aerogel within 54 s before the ending of illumination.
